# Tracing the scientific outputs in the field of Ebola research based on publications in the Web of Science

**DOI:** 10.1186/s13104-016-2026-2

**Published:** 2016-04-15

**Authors:** Fengyun Yi, Pin Yang, Huifeng Sheng

**Affiliations:** National Institute of Parasitic Diseases, Chinese Center for Disease Control and Prevention, Key Laboratory of Parasite and Vector Biology, Ministry of Health, WHO Collaborating Centre for Malaria, Schistosomiasis and Filariasis, Shanghai, 200025 China

**Keywords:** Bibliometrics, Ebola, ISI Web of Science

## Abstract

**Background:**

Ebola virus disease (hereafter EVD or Ebola) has a high fatality rate. The devastating effects of the current epidemic of Ebola in West Africa have put the global health response in acute focus. In response, the World Health Organization (WHO) has declared the Ebola outbreak in West Africa as a “Public Health Emergency of International Concern”. A small proportion of scientific literature is dedicated to Ebola research.

**Methods:**

To identify global research trends in Ebola research, the Institute for Scientific Information (ISI) Web of Science™ database was used to search for data, which encompassed original articles published from 1900 to 2013. The keyword “Ebola” was used to identify articles for the purposes of this review. In order to include all published items, the database was searched using the Basic Search method.

**Results:**

The earliest record of literature about Ebola indexed in the Web of Science is from 1977. A total of 2477 publications on Ebola, published between 1977 and 2014 (with the number of publications increasing annually), were retrieved from the database. Original research articles (*n* = 1623, 65.5 %) were the most common type of publication. Almost all (96.5 %) of the literature in this field was in English. The USA had the highest scientific output and greatest number of funding agencies. *Journal of Virology* published 239 papers on Ebola, followed by *Journal of Infectious Diseases* and *Virology*, which published 113 and 99 papers, respectively. A total of 1911 papers on Ebola were cited 61,477 times.

**Conclusion:**

This analysis identified the current state of research and trends in studies about Ebola between 1977 and 2014. Our bibliometric analysis provides a historical perspective on the progress in Ebola research.

**Electronic supplementary material:**

The online version of this article (doi:10.1186/s13104-016-2026-2) contains supplementary material, which is available to authorized users.

## Background

Ebola virus disease (hereafter EVD or Ebola) is a complex zoonosis that is highly virulent in humans. The largest recorded outbreak of EVD is ongoing in West Africa. It began in Guinea in December 2013 [1, 2] and has subsequently spread to Liberia, Sierra Leone, and Nigeria [3]. More people have been infected in this current outbreak than in all previous outbreaks combined. It is also the first outbreak that occurred in West Africa, which is outside the previously known range of the virus.

Since the first outbreak of Ebola in 1976 [4], there have been numerous other outbreaks in the human population across Africa, with case fatality rates typically around 60–70 %, but potentially reaching as high as 90 % [5]. Humans can become infected with Ebola after direct contact with the blood or bodily fluids of an infected person or animal. The virus also can infect and kill other primates, such as chimpanzees or gorillas, although *Pteropus giganteus* flying foxes are suspected to be the most likely carriers of the virus in the wild. The genus *Ebolavirus* comprises four species: *Zaire ebolavirus*, *Sudan ebolavirus*, *Reston ebolavirus*, and *Côte d’Ivoire* (*Ivory Coast*) *ebolavirus*. In 1994, a fifth species, *Taï Forest ebolavirus*, was isolated from a veterinarian who had autopsied a chimpanzee in Côte d’Ivoire [6], but the virus has not been detected subsequently. The final species, *Bundibugyo ebolavirus*, was responsible for an outbreak of EVD in Uganda in 2007 [7]. The virulence of the different Ebola viruses varies between strains. It is difficult to determine if the virus would infect and kill the same numbers of people in the developed world, which has better access to medical care. The provision of supportive care to Ebola-infected patients would likely lessen mortality; we can compare the 22 % case fatality rate seen in the Marburg virus outbreak in Germany with the 88–90 % mortality seen in the two major African outbreaks [8].

The devastating effects of the current epidemic of EVD in West Africa have put the global health response in acute focus. Fears of international spread of a Category A Priority Pathogen have made this a massive focus for international public health [9]. This has led to the current outbreak being declared a “Public Health Emergency of International Concern” by the WHO on 8 August 2014 [10–12].

The Institute for Scientific Information (ISI) Web of Science™ has a restriction rule for choosing the journal indexed in the database. Therefore, it is considered to be an authoritative database that scholars throughout the world can use. It is generally accepted that publications indexed in the Web of Science represent the central part of a research process. A large number of articles have been published since 1977 on Ebola. We conducted this study in an attempt to provide a bibliometric perspective of the progress in Ebola research using Web of Science.

## Methods

### Data source

Web of Science™ (Web of Knowledge) is an online subscription-based scientific citation indexing service maintained by Thomson Reuters (New York, NY, USA). It provides citation search, giving access to multiple databases that reference cross-disciplinary research and allowing for an in-depth exploration of specialized subfields [13]. We sought articles from Web of Science/Knowledge Citation Database. Two citation indexes, namely Science Citation Index Expanded and Social Sciences Citation Index^®^, were included. The keyword “Ebola” was used to identify papers for the purposes of this review. In order to include all published items, we used the Basic Search method to search Web of Science.

The output data showed that the earliest literature about Ebola indexed in the Web of Science was from 1977. The time period of “1977–2014” was considered to be long enough to retrieve a large number of articles. The impact factor (IF) was indexed in the ISI Journal of Citation Reports (JCR) ^®^ 2013.

This review is concerned with four types of papers on Ebola: articles, reviews, editorial materials, and news items. The fact that some article types attract more citations than others was taken into consideration. The retrieved papers were assessed using the following criteria: (1) publication year, (2) document type, (3) language(s), (4) countries/territories, (5) institution(s), (6) author(s), (7) research field, (8) journal of publication, and (9) number of citations. The search was done on January 31st, 2015.

### Data analysis

The analyses were performed using the analysis functions in Web of Science and the statistics functions in Microsoft Office^®^ Excel 2007. The results of the analyses are displayed in the figures and tables at the end of this paper. The journals’ IFs were evaluated using the JCR (Web of Science) 2012 edition, published by Thomson Reuters.

## Findings

### Annual output of papers about Ebola published between 1977 and 2014

**Fig. 1:**
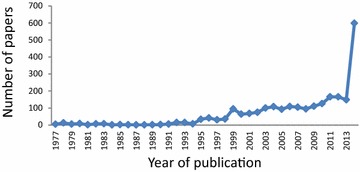
Output of papers about Ebola, published between 1977 and 2014, indexed in the Web of Science

A total of 2477 papers on Ebola, published between 1977 and 2014, were retrieved from Web of Science. From 1977 to 1994, the publication rate remained low, at about six articles per year. This was followed by several publication peaks in 1996, 1999, 2004, and 2011. The number of publications has been gradually increasing since 1995, when 34 papers were published, which was followed by a significant increase to 598 in 2014. The largest number of papers (*n* = 598, 24.1 %) was published in 2014, indicated that this field has been attracting more attention since the current Ebola outbreak. The increasing trend in the number of papers per year is illustrated in Fig. 1.

### Types and languages of papers about Ebola published between 1977 and 2014

The analysis of document types showed that original research articles (*n* = 1623, 65.5 %) were the most common type of papers about Ebola indexed in the Web of Science between 1977 and 2014. Other document types included news items (*n* = 295, 11.9 %), editorial materials (*n* = 294, 11.9 %), and reviews (*n* = 265, 10.7 %).

The analysis indicated that 96.5 % of the literature in this field was in English. The remaining publications were in Russian (*n* = 35, 1.4 %), French (*n* = 32, 1.3 %), and other languages. Other scholars have come to a similar conclusion that English is the dominant language of scientific papers, comprising 90–95 % of all Science Citation Index (SCI) papers [14, 15].

### Country/territory where published papers about Ebola came from between 1977 and 2014

**Fig. 2:**
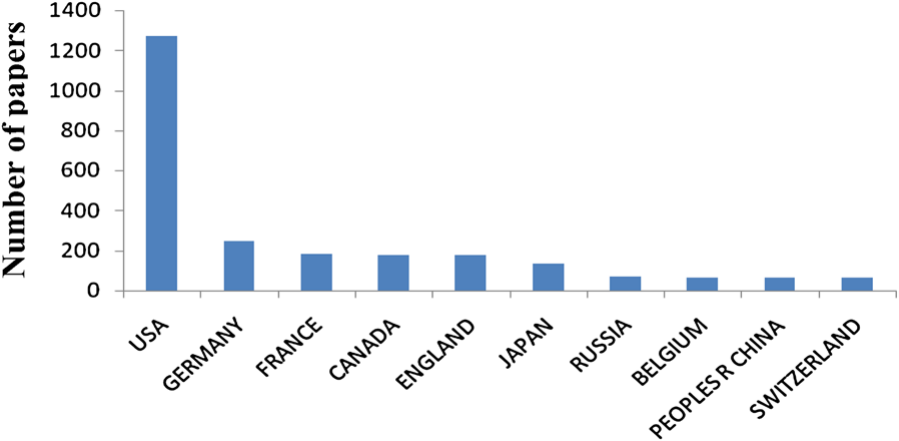
The top 10 countries/territories that published papers about Ebola, between 1977 and 2014, indexed in the Web of Science

Analysis of the contributions of different countries/territories to Ebola research was based on journal articles, in which addresses and affiliations of at least one author were provided. The USA has the highest scientific output worldwide with 1273 papers (51.4 %), followed by Germany (246 papers; 9.9 %) and France (183 papers; 7.4 %). China, with 62 papers (2.5 %), ranked 10th. The number of publications per country of the top 10 countries is illustrated in Fig. 2. Data for the other 62 countries/territories are not shown.

### Funding agency/institution supporting papers about Ebola published between 1977 and 2014

Our results show that 206 funding agencies financed 719 papers between 1977 and 2014. Each of the agencies funded three or more papers. Among the top 10 agencies, four were from the USA, funding a total of 360 papers (14.5 %), and two from Canada, funding a total of 41 papers (1.7 %). Good research and subsequent publications are dependent on funding support (see Table 1). Only eight papers (0.3 %) were supported by the National Natural Science Foundation of China.

**Table 1:** The top 10 funding agencies that supported/financed papers on Ebola, published between 1977 and 2014, indexed in the Web of Science

Funding agency/institution	Country	No. of papers	% of total publications
National Institutes of Health	USA	198	8.0
National Institute of Allergy and Infectious Diseases	USA	79	3.2
Defense Threat Reduction Agency	USA	62	2.5
Deutsche Forschungsgemeinschaft	Germany	40	1.6
Public Health Agency of Canada	Canada	26	1.0
National Science Foundation	USA	21	0.8
Canadian Institutes of Health Research	Canada	15	0.6
Ministry of Health Labor and Welfare of Japan	Japan	13	0.5
Agence Nationale de la Recherche	France	12	0.5
Japan Society for the Promotion of Science	Japan	11	0.4

### Authors of papers about Ebola published between 1977 and 2014

Overall, 1837 authors wrote the 2477 papers retrieved, giving an average of 1.3 authors per publication. H. Feldmann published 144 papers (5.8 %) and S. Becker published 72 papers (2.9 %)—which was much higher than any other author. The 10 most published authors wrote 698 papers (19.3 %), and were cited 32,849 times. The H-index was 79 (see Table 2). They were the main international cooperation teams in the field, which indicates an imbalance in the distribution of authors.

**Table 2:** The top 10 authors who wrote papers on Ebola, published between 1977 and 2014, indexed in the Web of Science

Authors	Institution	Number of papers published	Number of citations
Feldmann H	Laboratory of Virology, Division of Intramural Research, National Institute of Allergy and Infectious Diseases, National Institutes of Health, Hamilton, Montana, United States of America	144	4840
Becker S	Department of Nephrology, University Duisburg-Essen, Essen, Germany	72	2362
Bavari S	United States Army Medical Research Institute of Infectious Diseases, Fort Detrick, Frederick, USA	71	1936
Geisbert TW	Galveston National Laboratory and Department of Microbiology and Immunology, University of Texas Medical Branch, Galveston, TX, USA	71	4463
Jahrling PB	Integrated Research Facility at Fort Detrick, National Institute of Allergy and Infectious Diseases, National Institutes of Health, Fort Detrick, Frederick, Maryland, USA	63	4173
Rollin PE	Special Pathogens Branch, Centers for Disease Control and Prevention, Atlanta, Georgia	62	3709
Kawaoka Y	Division of Virology, Department of Microbiology and Immunology, Institute of Medical Science, University of Tokyo, Shirokanedai, Minato-ku, Tokyo, Japan (a); International Research Center for Infectious Diseases, Institute of Medical Science, University of Tokyo, Shirokanedai, Minato-ku, Tokyo, Japan (b); ERATO Infection-Induced Host Responses Project, Saitama, Japan (c); Department of Pathobiological Science, University of Wisconsin—Madison, Madison, Wisconsin, USA	58	2600
Klenk HD	Philipps-Universität Marburg, Institut für Virologie, Marburg, Germany	54	3816
Nichol ST	Viral Special Pathogens Branch, Division of High Consequence Pathogens and Pathology, Centers for Disease Control and Prevention, Atlanta, Georgia, USA	52	2711
Hensley LE	National Institutes of Health, National Institute of Allergy and Infectious Diseases, Integrated Research Facility, Fort Detrick, Maryland, USA	51	2239

### Research field of papers about Ebola published between 1977 and 2014

**Fig. 3:**
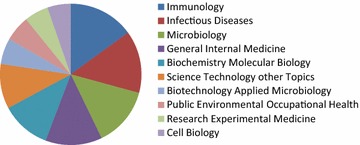
The top 10 research fields of papers about Ebola, published between 1977 and 2014, indexed in the Web of Science

Papers about Ebola indexed in the Web of Science were distributed over 67 research fields. The largest number of papers belonged to the virology field (680 papers; 27.45 %), followed by 334 papers (13.5 %) in the field of immunology, 318 papers (12.8 %) in the field of infectious diseases, and 303 papers (12.2 %) in the field of microbiology. Less than 300 papers belonged to other research areas. As a whole, the distribution of Web of Science categories showed that SCI papers concerned with Ebola mainly cover basic scientific research, with some dealing with clinical research (see Fig. 3).

### Journals in which papers about Ebola were published between 1977 and 2014

Our analysis showed that the 2477 retrieved papers were published in 263 journals. Among the journals publishing papers about Ebola between 1977 and 2014 that were indexed in the Web of Science, three journals had the highest numbers of papers about Ebola. *Journal of Virology* published 239 papers (9.6 %), followed by *Journal of Infectious Diseases* and *Virology*, which published 113 (4.56 %) and 99 (4 %) papers, respectively. Six out of the top 10 journals are from the USA, which is indicative of the country’s lead in Ebola-related research (see Table 3).

**Table 3:** The top 10 journals that published papers on Ebola between 1977 and 2014, indexed in the Web of Science

Journal	JCR^®^ category	Quartile in category	Publisher	IF	Research domain	No. of papers	% of total publications
Journal of Virology	Virology	Q1	USA	4.648	Virology	239	9.65
Journal of Infectious Diseases	Immunology, Infectious diseases, Microbiology	Q1	USA	5.778	Immunology, Infectious diseases, Microbiology	113	4.56
Virology	Virology	Q2	USA	3.278	Virology	99	4.00
British Medical Journal	Medicine, General & Internal	Q1	England	–	General & Internal Medicine	68	2.75
Science	Multidisciplinary Sciences	Q1	USA	31.477	Science & Technology—other Topics	56	2.26
Lancet	Medicine, General & Internal	Q1	England	39.207	General & Internal Medicine	54	2.18
Proceedings of the National Academy of Sciences of The United States of America	Multidisciplinary Sciences	Q1	USA	9.809	Science & Technology—other Topics	45	1.82
Nature	Multidisciplinary Sciences	Q1	England	42.351	Science & Technology—other Topics	43	1.74
Plos One	Multidisciplinary Sciences	Q1	USA	3.534	Science & Technology—other Topics	42	1.70
Viruses Basel	Virology	Q2	Switzerland	–	Virology	40	1.62

– Not found

### Number of citations in papers about Ebola published between 1977 and 2014

**Fig. 4:**
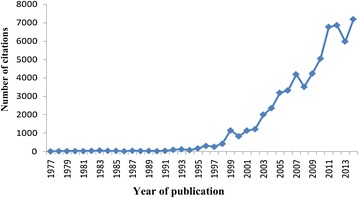
Citation of papers about Ebola, published between 1977 and 2014, indexed in the Web of Science

A total of 1911 papers on Ebola were cited 61,477 times in the Web of Science between 1977 and 2014. Until 1995, papers about Ebola were rarely cited. Since then, citations have consistently increased; papers about Ebola were cited 166 times in 1995. Other peaks occurred in 1996, 1999, 2007, 2012, and 2014 (see Fig. 4). The number of citations closely followed the number of publications. The number of citations per year reached its maximum in 2014 (7195 citations).

According to bibliometric (bibliometric methods are now firmly established as scientific specialties and are an integral part of research evaluation methodology, especially within the scientific and applied fields) principles, if a paper is cited more times than others, its quality is considered to be higher. Scientometrics (the study of measuring and analyzing science, technology, and innovation; the major research issues include the measurement of impact) has demonstrated that references are considered “classical references” once an article is cited four times or more [16]. The paper “Global trends in emerging infectious diseases” published in 2008 by the journal *Nature* has been cited the most (1065 times).

The top 50 most cited papers about Ebola have been cited more than 12,788 times. Each one has been cited 256 times on average. By definition, these have been classified as “classical references” in this field. Of these 50 most-cited papers, 10 were published in *Proceedings of the National Academy of Sciences of the United States of America*, and the remaining 40 were published in 20 different journals. Therefore, *Proceedings of the National Academy of Sciences of the United States of America* appears to be a key journal for publishing papers on Ebola. Of these 50 papers, eight were published each year between 2000 and 2003, and six were published each year between 2002 and 2004 (Additional file 1).

## Discussion

The objective of the present study was to perform a bibliometric analysis of all Ebola-related publications indexed in the Web of Science. Bibliometrics is a series of analyses used for evaluating or quantifying literature and information. It is a scientific method widely used in many fields. All Science Citation Index papers were analyzed for various factors.

This analysis determined the current state of research and trends in studies about Ebola between 1977 and 2014. The number of papers has gradually been increasing over the 38-year study period, especially since early 1995. The increasing publication output may be attributed to the isolation of the *Taï Forest ebolavirus* strain in 1994. Five publication peaks were noted, in 1996, 1999, 2004, 2011, and 2014. Each peak can be directly affiliated with a historical event.

In 1976, the reappearance of hemorrhagic fever outbreaks, caused by a distinct species of the *Filoviridae* family, affected people in South Sudan and Zaire [17, 18]. The *Ebolavirus* genus is named after the Ebola river, a tributary of the Congo River and an area where the first documented modern case of infection was identified in 1976 [19]. Therefore, it makes sense that the earliest literature about Ebola retrieved from Web of Science was published in 1977. Since then, Ebola outbreaks have occurred from time to time in Africa. So far, there have been 19 outbreaks, primarily in central African countries including the DRC (1977, 1995, 2007, 2008, and 2012); Sudan (1976, 1979, and 2004); Gabon (1994, 1996, 2001, and 2002), Uganda (2000, 2007, and 2012); and the Congo (2001–2002, 2003, and two in 2005). Two thousand four hundred and three patients have been affected and 1594 patients have died from Ebola (66.3 %) [20]. In August 2014, the largest, most sustained, and widespread Ebola outbreak in history was declared to be a “Public Health Emergency of International Concern” by the WHO [21, 22]. These events lead to a rise in the number of publications related to Ebola.

Almost two-thirds (65.5 %) of the retrieved papers about Ebola were original research articles. Almost all (96.5 %) of the literature in the field was in English. For better international communication, English is the first language of choice for many authors. It was found that the USA has the highest scientific output worldwide, publishing 1273 papers about Ebola. The country also dominates in other clinical disciplines, such as urology [23], orthopedic surgery [24], critical care medicine [25], general surgery [26], emergency medicine [27], and anesthesia [28]. Beyond doubt, the USA is a key player in the field of Ebola research.

The top three journals that published papers on Ebola between 1977 and 2014 were *Journal of Virology*, *Journal of Infectious Diseases*, and *Virology*. Among the top 10 journals, six were from the USA. All top 10 journals were from developed countries. In the field of Ebola, developed countries have had a great influence on high-level science and the development of technology. Furthermore, publications from these journals were of high quality. The underlying reasons for these findings could be attributed to the USA’s large population of senior researchers and adequate research budgets for scientific investigations. There is obviously a long way to go for other countries to catch up.

The analysis of journals in which papers about Ebola were published could help scholars select the appropriate journal for paper submission, thereby increasing the chance of acceptance. The analysis of the authors who published the most papers about Ebola could, to some degree, help encourage global cooperation and teamwork in the field of Ebola research. It can help researchers make the best use of available resources to increase efficiency and accelerate progress. The highly-cited articles and reviews are “classical” papers that students and primary researchers should read carefully, particularly if they plan to devote themselves to this field. The average number of citations is very high; the most-cited paper was cited 1065 times (up-to-date the data were downloaded), indicating that people are paying more attention to Ebola.

Our review had several limitations. Our search of the Web of Science expanded database from 1970 to 2014 is quite sensitive and papers published elsewhere were excluded, so it is likely that some true “classic” articles were missed. We believe that there are many research prospects in this area.

## Additional file

10.1186/s13104-016-2026-2 Highly cited papers on Ebola in Web of Science during 1977–2014.

## Abbreviations

EVD: Ebola virus disease
IF: impact factor
ISI: Institute for Scientific Information
JCR: Journal Citation Reports^®^
WHO: World Health Organization
